# Diagnosis and management of invasive candidiasis in the ICU: an updated approach to an old enemy

**DOI:** 10.1186/s13054-016-1313-6

**Published:** 2016-05-27

**Authors:** Thierry Calandra, Jason A. Roberts, Massimo Antonelli, Matteo Bassetti, Jean-Louis Vincent

**Affiliations:** Infectious Diseases Service, Centre Hospitalier Universitaire Vaudois, University of Lausanne, Rue du Bugnon 46, 1011 Lausanne, Switzerland; Burns, Trauma and Critical Care Research Centre, The University of Queensland, Royal Brisbane and Women’s Hospital, Butterfield Street, 4029 Herston, Brisbane, Australia; Department of Anesthesiology and Intensive Care Medicine, Catholic University of Rome, A. Gemelli University Hospital, Largo A. Gemelli 8, 00168 Rome, Italy; Infectious Diseases Division, Santa Maria Misericordia University Hospital, Piazzale Santa MAria della Misericordia 15, 33100 Udine, Italy; Department of Intensive Care, Erasme Hospital, Université libre de Bruxelles, Route de Lennik 808, 1070 Brussels, Belgium

## Abstract

Invasive fungal infections, particularly those caused by *Candida* species, are not uncommon in critically ill patients and are associated with considerable morbidity and mortality. Diagnosis and management of these infections can be challenging. In this review, we will briefly discuss recent epidemiological data on invasive candidiasis and current diagnostic approaches before concentrating on antifungal treatments.

## Background

Invasive fungal infections in critically ill patients are associated with considerable morbidity and mortality. *Candida* and *Aspergillus* species are the most frequent causes of healthcare-associated fungal infections in these patients [1]. Although *Candida* infections are the most frequent fungal infections in ICU patients, invasive aspergillosis is associated with higher morbidity and mortality rates, even in the absence of traditional hematological risk factors [2]. Occasionally, cryptococcosis, pneumocystosis, or zygomycosis may also be encountered in the ICU setting in patients with solid organ or hematopoietic stem cell transplantation, acquired immunodeficiency syndrome, cancer, or hematological malignancies. Because *Candida* species account for 70–90 % of invasive fungal infections, this overview will focus on invasive candidiasis and the important aspects that must be considered as part of optimizing treatment.

## Epidemiology

In the USA, *Candida* species are responsible for 8–10 % of bloodstream infections (BSI) and are the fourth most common bloodstream pathogen. In Europe, *Candida* species only account for 2–3 % of BSI and are ranked as the 6th–10th most frequent pathogen [3]. Approximately 30–35 % of all episodes of candidemia occur in ICU patients [4]. In the Extended Prevalence of Infection in Intensive Care (EPIC II) point prevalence study [5], *Candida* was isolated in 17 % of infected culture-positive patients. Several studies have reported increasing incidence rates of candidemia in ICUs over recent years [6, 7], although population-based studies have suggested reduced incidence rates possibly related to improved infection control practices in high-risk patients [8, 9].

Invasive candidiasis is a highly lethal infection associated with mortality rates between 40 and 60 % [6, 7, 10, 11]. The five most common *Candida* species are *Candida albicans*, *Candida glabrata*, *Candida tropicalis*, *Candida parapsilosis*, and *Candida krusei* [12]. *C. albicans* was previously the predominant species isolated in patients with invasive candidiasis, accounting for 65–70 % of the total number of *Candida* isolates. However, the epidemiology has changed in recent years, with non-*albicans* species now responsible for about half of the cases in some centers [10, 13, 14]. *C. parapsilosis* tends to be more frequent in southern Europe, Australia, and Latin America than elsewhere [10, 15–17], and *C. glabrata* is more frequent in the older population than in middle-aged adults [7, 15]. Non-*albicans* species often have reduced susceptibilities or even intrinsic resistance to azoles or echinocandins [17]. These epidemiological trends underscore the need to perform large longitudinal studies to obtain data that can be used to guide empirical antifungal therapy.

## Diagnosis

Prompt and accurate diagnosis of invasive fungal infection is crucial so that appropriate antifungal agents can be started rapidly. However, early diagnosis is not always easy. Microscopic examination is rapid and can be helpful but a negative result does not exclude infection [18, 19]. Blood cultures are positive in only 50–70 % of cases of *Candida* BSI [20]. Furthermore, it can take several days before *Candida* is identified at the species level and antifungal susceptibility data are available. Moreover, blood cultures are rarely positive in patients with deep-seated candidiasis [20]. In such patients, cultures of infected tissues can be performed but have their own limitations, including the need for invasive surgical procedures and poor sensitivity [20].

Recently, nonculture-based diagnostic tests have been developed for detection in blood of components of the fungal cell wall (such as mannan and β-d-glucan (BDG)) by immunoassays, of DNA by PCR, and of antibodies by serology. The performance of BDG assays in invasive fungal infections has been assessed in three systematic reviews. In the subgroup of patients with proven infection, the pooled sensitivities and specificities of BDG were 79.1, 87.7, and 78 %, respectively [21–23]. Importantly, BDG assays are not species specific so further tests are needed to identify the specific fungus. In patients with suspected invasive candidiasis, the presence of elevated circulating levels of *Candida* mannan antigen in the blood had a sensitivity of 58 % and a specificity of 93 % [24]. In the same group of patients, the detection of anti-mannan antibodies had a sensitivity of 59 % and a specificity of 83 %. The sensitivity increased to 83 % when the mannan and anti-mannan assays were combined, but the specificity remained similar at 86 %. PCRs for fungi continue to be challenging for technical reasons and there are no commercially available tests at present. In a review of more than 50 standard, nested, or real-time PCRs [25], the pooled sensitivity and specificity were 95 and 92 %, respectively, for invasive candidiasis.

Combining several markers may prove more useful. A technology combining PCR and nanoparticle-based hybridization, the T2 magnetic resonance-based biosensing technology platform, can detect as little as 1 colony-forming unit/ml of the five most common *Candida* species [26] and was shown to have an overall specificity of 99.4 % for invasive candidemia [27]. This test is expensive to perform, however, and although costs may be balanced by more accurate earlier diagnosis with reduced administration of antifungal agents and reduced mortality rates [28], further studies are needed to validate its use and assess its cost–benefit ratio.

## Risk factors

Numerous risk factors for invasive *Candida* infections have been identified, including higher Acute Physiology and Chronic Health Evaluation II scores, diabetes mellitus, renal insufficiency, surgery (especially abdominal surgery), pancreatitis, the use of broad-spectrum antibiotics, parenteral nutrition, hemodialysis, mechanical ventilation, the presence of central vascular catheters, and therapy with immunosuppressive agents [29–34]. The development of invasive *Candida* infections is often preceded by extensive colonization of the skin or of the mucus membranes of the gastrointestinal and urogenital tracts, and the degree of colonization, assessed using the Colonization Index, has been shown to be an independent risk factor for development of candidiasis [35, 36].

Several prediction rules and scores based on clinical, laboratory, and microbiological parameters have been proposed to help clinicians identify patients at high risk of developing invasive fungal infections [32, 33, 35, 37–41]. Ostrosky-Zeichner et al. [40] proposed a prediction rule characterized by a very high negative predictive value (0.97) and including the following parameters: use of systemic antibiotic therapy, total parenteral nutrition, dialysis, steroids or immunosuppressive agents, major surgery or pancreatitis, and the presence of a central venous catheter. The Candida score, an easy-to-use assessment system proposed by Leon et al. [32, 33], integrates four risk factors (total parenteral nutrition, surgery, multifocal *Candida* colonization, and severe sepsis) and also has a high negative predictive value (0.98) to rule out invasive candidiasis [33]. However, colonization surveillance cultures are expensive and their precise value remains unclear [42].

More recently, biomarkers of fungal infection have been suggested to assist in decisions to start or stop antifungals. BDG was superior to the Colonization Index or the Candida score for the prediction of intraabdominal candidiasis in high-risk surgical and ICU patients, with a cutoff value of 80 pg/ml [43]. Similarly a prospective observational study demonstrated that BDG was more accurate than the Candida score and the Colonization Index for early prediction of invasive *Candida* infection in patients at risk for *Candida* sepsis [44], with 250 pg/ml identified as the best cutoff value [44]. A decrease in BDG levels during antifungal treatment for invasive candidiasis correlated with a successful clinical response with a positive predictive value of 90 % [45]. However, despite its promising performance for excluding invasive infection and orienting the duration of therapy, the cost of the BDG test and a lack of large validation studies currently limit its use.

## Management

Antifungal treatment can be considered as prophylactic (in patients at high risk of fungal infection), empiric (triggered by clinical signs of fungal infection, e.g., persistent fever in the presence of risk factors), preemptive (triggered by microbiological or biomarker evidence of fungus without actual infection), and definitive (after positive microbiological confirmation of strain and sensitivity of fungus).

Prophylactic antifungal agents are recommended in just a few specific situations and most treatments are given on an empiric basis [46]. Although use of preemptive therapy is gaining interest, more studies are needed to better define which patients may benefit from this approach [47] and whether more widespread use of antifungal agents may negatively influence fungal ecology [48]. In a recent study of 241 ICU patients requiring emergency gastrointestinal surgery for intraabdominal infection, preemptive therapy with micafungin was not effective at reducing the development of invasive candidiasis compared with placebo, although there was some suggestion that this may have been the result of the preemptive therapy being administered too late [49].

There are three main groups of antifungals: the azoles, the polyenes, and the echinocandins. The selection of an antifungal regimen is based on multiple factors, including epidemiological data, patient characteristics, hospital setting, fungal strain, site of infection, and safety profiles of the antifungal agents. Although different antifungals can show comparable efficacy in treating candidemia, their differences in terms of pharmacokinetics (PK) and pharmacodynamics (PD) (including fungistatic versus fungicidal activity, need for dose adjustment in case of hepatic or renal failure and drug–drug interactions), toxicity, and selection pressure remain significant and can affect the clinical outcome of fragile patient populations, such as the critically ill. Specific guidelines are therefore available to help direct optimal antifungal choices [12, 46, 50]. The European Society of Clinical Microbiology and Infectious Diseases (ESCMID) guidelines no longer consider fluconazole the drug of choice for invasive candidiasis, and endorse the use of echinocandins as first-line empiric treatment [46]. The rationale for this statement is that, compared with fluconazole, echinocandins show a broader spectrum of activity, fungicidal activity, an excellent safety profile, and fewer drug–drug interactions. The recent Infectious Diseases Society of America (IDSA) guidelines also recommend echinocandins for initial therapy [12]*.* All three available echinocandins (caspofungin, micafungin, and anidulafungin) penetrate well into the biofilm formed on vascular devices. Nevertheless, fluconazole remains a well-known and well-tolerated antifungal with a significantly lower cost compared with the echinocandins and may still be of value in clinically stable patients who have had no recent azole exposure or have known fluconazole-sensitive organisms [12].

### Dosing of antifungal agents

Pathophysiological changes associated with critical illness can change drug concentrations so that they are significantly different from those observed in noncritically ill patients. In these circumstances, if standard dosing is used, then suboptimal concentrations (either too low or unnecessarily high) may result, putting the patient at risk of clinical failure or drug toxicity. Antifungal agents tend not to be as markedly affected by altered PK in critical illness. The drugs are mostly lipophilic, hepatically metabolized, and with high protein binding. Drugs such as fluconazole are an exception to these characteristics. Use of renal replacement therapy (RRT) can influence the clearance of some antifungal drugs, particularly those that are predominantly eliminated by renal mechanisms, are not highly protein bound, and have low molecular weights, notably the azoles and 5-flucytosine. Moreover, different RRT characteristics, including the specific mode, membrane characteristics, flow rates, and duration of treatment, may influence the effects of RRT on drug PK [51] and should be taken into consideration when evaluating dosing.

From a PD perspective, efficacy for the triazole antifungals is described in terms of the ratio of the area under the concentration–time curve (AUC) to the minimum inhibitory concentration (MIC). The echinocandins are mostly described in terms of the ratio of the maximum concentration in a dosing interval (Cmax) to the MIC. Fluconazole is the antifungal for which most of the dosing challenges for critically ill patients exist. Data comparing the PK of fluconazole in critically ill patients and noncritically ill patients show a lower AUC, including both Cmax and minimum concentration during a dosing interval [52]. Lack of achievement of the AUC/MIC target in particular is associated with doses <6 mg/kg, suggesting that standard maintenance dosing should be at this level. In sepsis, interstitial fluid concentrations of fluconazole in tissues, which are commonly the target site of infection, are lower than those in plasma by ~50 % [53]. These data suggest that reduced efficacy may occur in states of microvascular failure. In the presence of RRT, very high drug clearances of fluconazole are common, necessitating even higher doses than in patients with normal renal function. Few data are available regarding altered PK of posaconazole, itraconazole, or isavuconazole in critically ill patients. For voriconazole, the unpredictability of dosing for individual patients tends to mean that critical illness does not in itself result in differences to dosing recommendations—although issues such as drug–drug interactions may be more common in these patients, necessitating more careful dose considerations.

There are only sparse data to suggest that the PK of echinocandins changes significantly in critically ill patients and they are not greatly influenced by RRT. A reduced AUC has been described for anidulafungin and caspofungin with a lower Cmax [52], but this is yet to be correlated with reduced efficacy. Use of adequate loading doses is particularly important for these agents (except micafungin), with caspofungin not even reaching steady-state concentrations by day 3 of treatment [54]. The high protein binding of echinocandins may mean that the presence of hypoalbuminemia could severely alter the PK. Preliminary data for micafungin demonstrate an altered volume of distribution with changes in serum albumin concentrations [55]. There are again few data for the various amphotericin formulations or flucytosine. Table 1 presents a summary guidance based on current understanding of how doses should be adjusted in different subpopulations of critically ill patients. Therapeutic drug monitoring (TDM) is likely to play an increasingly important role in our antifungal dosing decisions, and recommendations for when TDM should be used with the different agents available have been published recently [56]. To the best of our knowledge, there are no studies in critically ill patients with invasive fungal infections that have measured the patient outcome benefits of a dose-optimization approach, such as TDM. Until such data become available, results from other patient populations, which support achievement of PK/PD targets and the use of TDM, suggest that we should actively optimize antifungal dosing, including the use of TDM where available [57–60].

**Table 1:** Summary guidance for antifungal dosing in different critically ill patient subpopulations

	ARC	AKI	RRT	ALF
Amphotericin	Unchanged	Unchanged	Unchanged	Unchanged
Fluconazole	Increase	Decrease	Increase	? Unchanged
Voriconazole	TDM	TDM	TDM	TDM
Itraconazole	TDM	TDM	TDM	TDM
Posaconazole	TDM	TDM	TDM	TDM
Caspofungin	Unchanged	Unchanged	Unchanged	Decrease
Micafungin	Unchanged	Unchanged	Unchanged	Unchanged
Anidulafungin	Unchanged	Unchanged	Unchanged	Unchanged
Flucytosine	TDM	TDM	TDM	TDM

*AKI* acute kidney injury, *ALF* acute liver failure, *ARC* augmented renal clearance, *RRT* renal replacement therapy, *TDM* therapeutic drug monitoring

## Conclusion

Fungal infections are associated with considerable morbidity and mortality in ICU patients. Therapies are often started late because these infections can be difficult to diagnose. Until rapid susceptibility testing is available, empiric therapy should be used based on known patient characteristics, including risk factors, local fungal microbiology patterns, hospital setting, and site of infection. Further data from epidemiological studies are needed to help better define high-risk populations, and thus guide empirical antifungal choices. Further development of PCR and other diagnostic and predictive tests will probably lead to increased use of preemptive therapy, but studies are needed to confirm the beneficial effects of preemptive therapy on outcomes before this approach can be recommended. Dosing needs are different in ICU compared to non-ICU patients but data remain limited, with current evidence suggesting that dosing should be individualized according to patient characteristics in order to ensure optimal outcomes.

## Abbreviations

AUC: Area under the concentration–time curve
BDG: β-d-Glucan
BSI: Bloodstream infections
Cmax: maximum concentration in a dosing interval
MIC: minimum inhibitory concentration
PD: Pharmacodynamics
PK: Pharmacokinetics
RRT: renal replacement therapy
TDM: therapeutic drug monitoring
